# *Mycobacterium tuberculosis* multistage antigens confer comprehensive protection against pre- and post-exposure infections by driving Th1-type T cell immunity

**DOI:** 10.18632/oncotarget.11542

**Published:** 2016-08-23

**Authors:** Jilei Ma, Maopeng Tian, Xionglin Fan, Qi Yu, Yukai Jing, Weihua Wang, Li Li, Zijie Zhou

**Affiliations:** ^1^ Department of Pathogen Biology, School of Basic Medicine, Tongji Medical College, Huazhong University of Science and Technology, Wuhan 430030, People's Republic of China; ^2^ Wuhan Pulmonary Hospital, Wuhan Institute for Tuberculosis Control, Wuhan 430030, People's Republic of China

**Keywords:** TB, subunit vaccine, primary infection, post-exposure, Th1

## Abstract

There is an urgent need for a vaccine against tuberculosis (TB) that is more effective than the current sole licensed option. However, target antigens of *Mycobacterium tuberculosis* with the vaccine potential remain elusive. Five immunodominant antigens with characteristic expressions at the stages of primary infection (Ag85A), the regulation of nutrition and metabolism when transferring from rapid growth to latency (PhoY2 and Rv3407), latency (Rv2626c), and reactivation (RpfB) were selected to construct the fusion polyprotein WH121, which has better immunogenicity and protection than each multistage antigen. DMT adjuvanted WH121 vaccinated C57BL/6 mice could confer persistent and significant protection against the respiratory challenge with 80 CFU of virulent *M. tuberculosis* H37Rv at 9 and 18 weeks after immunization, as the BCG vaccine did. Moreover, WH121/DMT could boost the BCG primed mice against post-exposure infection, and more significantly inhibit the growth of *M. tuberculosis* in the spleen than BCG repeat vaccination. The protection elicited by WH121/DMT is attributed to the WH121-specific Th1-type biased immune responses, characterized by increased antigen-specific IgG2a/IgG1 ratio and high levels of IFN-γ secreted by the splenocytes of vaccinated mice. In particular, high levels of IFN-γ^+^ T_EM_ cells in the spleen are an effective biomarker for the vaccine-induced early protection, and the persistent protection mainly depends on the increasing IL-2^+^IFN-γ^+^CD4^+^ and CD8^+^ T cells, especially IL-2^+^ T_CM_ cells. These findings demonstrate that multistage-specific antigens might be promising targets for the next generation TB vaccine, and a combination of these antigens such as WH121/DMT is required for further preclinical evaluation.

## INTRODUCTION

Despite the fact that there is currently only one licensed vaccine, *Mycobacterium bovis* Bacillus Calmette-Guerin (BCG), against tuberculosis (TB). It is used worldwide [1] and effectively protects children from primary TB [2]. However, it exhibits highly variable efficacy against adult TB [3], and most of TB's morbidity and mortality are associated with adult infections. TB remains the leading cause of death among infectious diseases, and HIV co-infection with TB further exacerbates the threat. As estimated by the World Health Organization (WHO) in 2014, approximately 12% of 9.6 million new TB cases were HIV positive, which resulted in 1.5 million deaths including 0.4 million HIV infected persons globally [4]. To conquer TB, there is an urgent need to propose a novel strategy to exploit a more efficacious vaccine.

For safety reasons, TB subunit vaccines have attracted much more attentions in the past few years. The clinical features of TB infection include multistage processes of primary infection, latency and reactivation. *In vitro* models of *M. tuberculosis* to mimic environmental conditions of different stages *in vivo* have demonstrated expression of differential antigens in *M. tuberculosis* under these situations. Correspondingly, early vaccine candidates were constructed based on the antigens that are secreted by rapidly growing *M. tuberculosis* during acute primary infection, such as the fusion proteins Ag85B-ESAT-6 and Ag85B-TB10.4 in clinical trials [5] and many fusion or mixed proteins in preclinical development. Few studies have reported on further two-stage antigen-based TB subunits such as the fusion proteins of both latent and secreted antigens H56 (Ag85B-Rv2660c-ESAT-6) [6] or ID93 (Rv2608-Rv3619-Rv3620-Rv1813) [7] in clinical trials, or in the preclinical investigation of Mtb10.4-HspX [8], ESAT6-RpfE [8], the poly-epitope protein derived from the Hsp65-Ag85B-19 kDa lipoprotein-Hsp16 and Rv1733c [9]. However, the protection induced by these novel TB vaccine candidates is very difficult to reach or surpass that of BCG, because BCG is a live attenuated vaccine that can express and secrete more than thousands of proteins. Although the general trend is the combination of many more antigens for TB vaccine candidates, the suitable antigens as vaccine targets from multistage expressions of *M. tuberculosis in vivo* remain unclear, which is a huge obstacle when developing an effective vaccine.

In our recent study, the high frequency of responders to both Ag85B and other four latent antigens (namely, Rv1813, Rv2660c, Rv2623, and HspX) were found in both active TB patients (ATBs) and latent tuberculosis infections (LTBIs) [10], which suggests that both replicated and latent *M. tuberculosis* might coexist in a presumptive host after infection. Considering the multistage process of TB pathogenesis, we assume that a subunit protein vaccine based on a combination of the more stage-specific immunodominant antigens in *M. tuberculosis* could provide more comprehensive protection against TB infection.

In this study, five selected antigens including Rv3407, PhoY2, Ag85A, Rv2626c and RpfB, which were characteristically expressed during different stages of primary infection, latency, regulation from the rapid growth to latency, and reactivation of *M. tuberculosis*, were selected to construct a multistage polyprotein called WH121. The immunogenicity and protective efficacy of WH121 in adjuvant DMT [11] was evaluated in C57BL/6 mouse models as pre- and post-exposure vaccines, respectively.

## RESULTS

### Purification and identification of recombinant WH121 and multistage antigens

**Figure 1:**
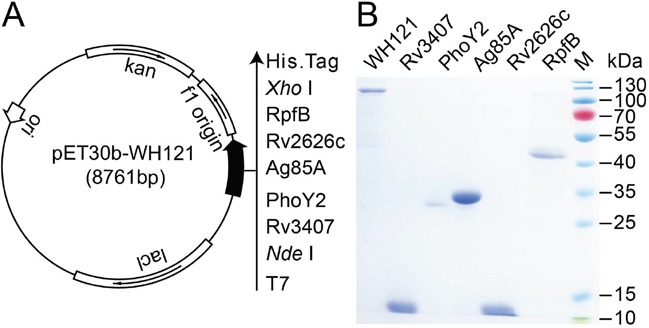
Purification and identification of WH121 and multistage antigens **A.** The recombinant prokaryotic expression plasmid pET30b-WH121. **B.** The identification of purified WH121 and multistage antigens Rv3407, PhoY2, Ag85A, Rv2626c, and RpfB by 15% SDS-PAGE.

The tandem-linking DNA sequence encoding five *M. tuberculosis* antigens Rv3407, PhoY2, Ag85A, Rv2626c, and RpfB, was successfully designed and synthesized, and then constructed as the recombinant prokaryotic expression plasmid pET30b-WH121 (Figure 1A), which was confirmed by enzyme digestion and DNA sequencing. The recombinant polyprotein WH121 with its C-terminal His-tag was efficiently expressed in the pET30b-WH121-transformed *E. coli* BL21(DE3) strain as inclusion bodies, and purification was performed under denaturing conditions. The multistage antigens Rv3407, PhoY2, Rv2626c, and RpfB that were also constructed with a C-terminal His-tag were effectively expressed in their respective recombinant *E. coli* strains. The recombinant protein Ag85A was prepared as previously described [12]. A single major band with the expected molecular weight was revealed by SDS-PAGE analysis for all purified proteins and the purified WH121 polyprotein of 121 kDa also had a high degree of purity (Figure 1B).

### Stronger immunogenicity and better protection of WH121 than single multistage antigens

**Figure 2:**
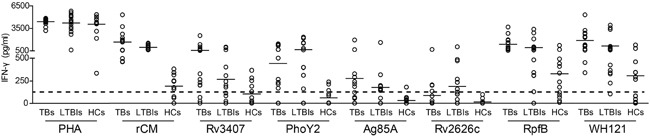
Comparison of the immunogenicity of WH121 and multistage antigens Whole blood samples were obtained from each member of ATBs (n=15), LTBIs (n=16), and HCs (n=13) and stimulated with 20 μL of either WH121, Rv3407, PhoY2, Ag85A, Rv2626c, RpfB (each 10 μg), or saline for 18 to 24 h. Each spot represents the antigen-specific concentration of IFN-γ in a sample, which was determined with a commercial ELISA kit. The median values for the different groups are indicated by horizontal lines and the dotted line represents the cut-off value for a positive response to each multistage antigen, which was set arbitrarily at 3X the mean of the negative control value for HCs.

To compare the immunogenicity of the polyprotein WH121 and each multistage antigens, 15 ATBs, 16 LTBIs, and 13 non-infected healthy controls (HCs) were screened and confirmed based on the default diagnostic criteria, and whole blood IFN-γ release assay (WBIA) was performed. There was no significant difference among the three groups in terms of the level of IFN-γ detected in the PHA-WBIA positive control. Among all five multistage antigens, Rv2626c, Ag85A, PhoY2, and Rv3407 induced low IFN-γ responses in HCs with 0, 7.7%, 15.4% and 30.8% responders, respectively. In contrast, multistage *M. tuberculosis* antigens were more easily recognized by T cells from both LTBIs and ATBs than HCs. The proportions of responders per multistage antigens in the ATBs and LTBIs were as follows: Rv2626c, 20% versus 37.5% (*p* = 0.227); Ag85A, 46.7% versus 43.8% (*p* = 0.534); PhoY2, 66.7 % versus 62.5% (*p* = 0.416); and Rv3407, 80 % versus 56.3% (*p* = 0.134). The highest frequency recognized by T cells was RpfB, which was the strongest immunogenicity of all five multistage antigens in three groups, increasing from 61.5% of the HCs to 81.3% and 100% of the LTBIs and ATBs. Most importantly, the fusion protein of WH121 in different populations also had better immunogenicity than single multistage antigen except RpfB. 53.8% of the HCs, 93.8% of the LTBIs and 100% of the ATBs reacted against WH121 (Figure 2).

**Figure 3:**
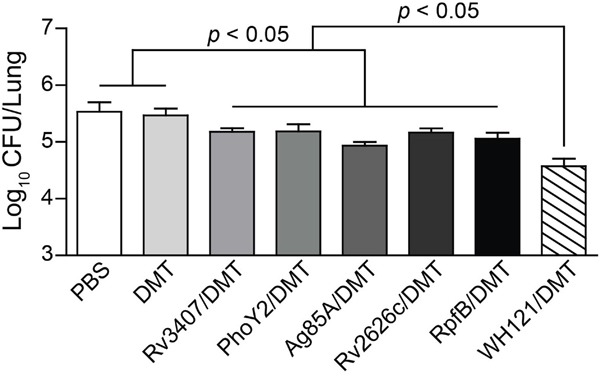
Comparison of the protection of WH121 and multistage antigens C57BL/6 mice were vaccinated with PBS, DMT, Rv3407/DMT, PhoY2/DMT, Ag85A/DMT, Rv2626c/DMT, RpfB/DMT or WH121/DMT, respectively. Six weeks later, vaccinated C57BL/6 mice were challenged i.n. with 80 CFU of the *M. tuberculosis* H37Rv. Four weeks post-challenge, their lungs were harvested, and the CFU numbers per lung were counted. The results were shown as the mean of log_10_ CFU ± SEM per lung for each group (n=6).

Moreover, the protection conferred by DMT adjuvanted WH121 or single multistage antigen was evaluated in vaccinated C57BL/6 mice by challenged intranasally (i.n.) with approximately 80 CFU of *M. tuberculosis* at six weeks after immunization. Four weeks later, the bacterial load in the lung was compared between different groups. Of all groups, PBS control mice had the highest bacterial load of *M. tuberculosis* in the lung of the infected mice (*p* < 0.05, Figure 3). Most importantly, vaccinating with WH121/DMT more significantly inhibited the growth of *M. tuberculosis* in the lung of infected C57BL/6 mice than both PBS control and each multistage antigen groups, although mice vaccinated with single multistage antigen also had a lower lung bacterial load than PBS control (*p* < 0.05).

### Th1-type biased responses induced by WH121/DMT

**Figure 4:**
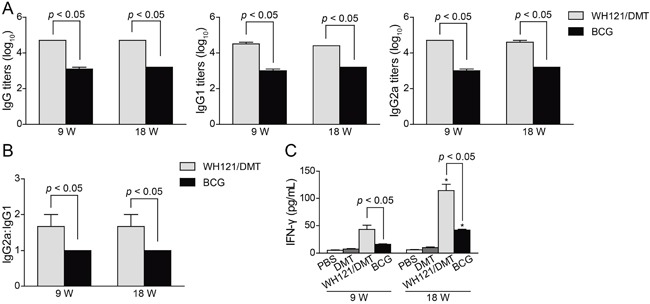
Th1 biased immune responses to WH121 in immunized mice (n=6) 9 and 18 weeks after immunization, sera were collected from each C57BL/6 mouse vaccinated with WH121/DMT, BCG, DMT, or PBS. **A.** The IgG, IgG1, and IgG2a antibodies against WH121 in the immunized mice were detected by ELISA. The results are shown as the mean (±SEM) log_10_ endpoint titer and **B.** the ratio of IgG2a:IgG1 in the differently vaccinated mice. **C.** WH121-specific IFN-γ levels secreted by the splenocytes of different vaccinated mice. Splenocytes were obtained from each mouse in the different vaccinated groups at 9 and 18 weeks after immunization. A 2.5×10^6^ cell amount was added to each well in the 24-well microtiter plates and incubated with WH121 protein (10 μg/mL) for 72 h at 37°C and 5% CO_2_. The IFN-γ concentrations in the suspension were detected with a commercial ELISA kit. The results are shown as the mean ± SD (pg/mL). *p < 0.05, 9 vs. 18 weeks. Similar results were obtained from two independent experiments.

To evaluate the immunogenicity of WH121 in the DMT adjuvant in C57BL/6 mice, WH121-specific antibodies, including IgG, IgG1, and IgG2a, were determined by ELISA at 9 and 18 weeks after immunization (Figure 4A). As expected, there was no induction of these antibodies in the PBS control and DMT groups (data not shown). Mice vaccinated with WH121/DMT exhibited significantly higher levels of WH121-specific IgG, IgG1, and IgG2a antibodies than the BCG group (*p* < 0.05), although each antibodies against the WH121 polyprotein were kept stable over time in both groups. Moreover, the ratio of IgG2a/IgG1 in the WH121/DMT group was also much higher than that of the BCG group during the entire experimental period (Figure 4B).

Furthermore, the WH121-specific Th1-type cytokine IFN-γ secreted by splenocytes from different groups were also compared (Figure 4C). Of all groups, the lowest level of WH121-specific IFN-γ was secreted by splenocytes from PBS control mice as expected at 9 and 18 weeks after immunization (*p* < 0.05). Although the levels of WH121-specific IFN-γ were increased over time (*p* < 0.05) in both groups, WH121/DMT induced a higher level of IFN-γ response to WH121 than the BCG vaccine (*p* < 0.001).

### Higher levels of effector and central memory T cells elicited by WH121/DMT

**Figure 5:**
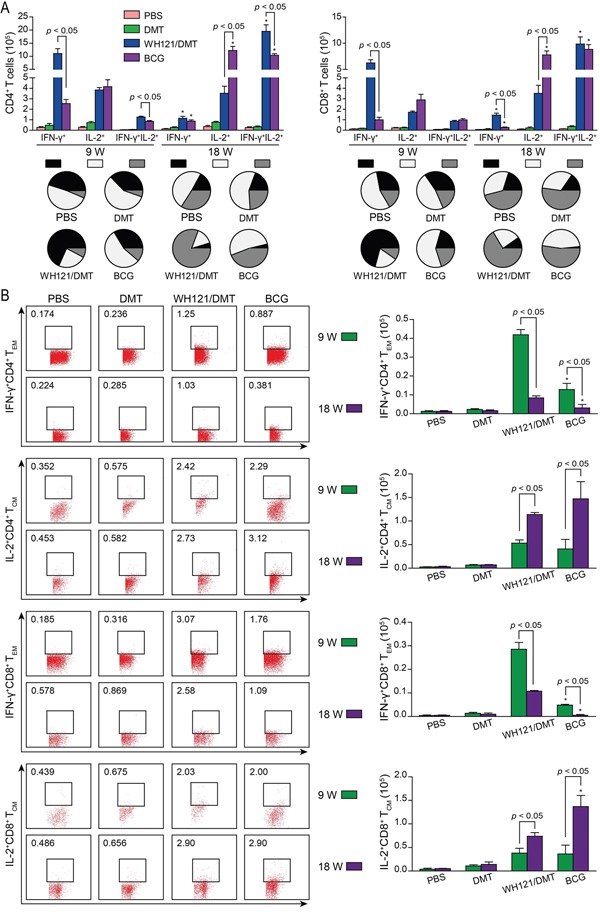
WH121-specific effector and central memory T cells 9 and 18 weeks after immunization, splenocytes were prepared from each C57BL/6 mouse vaccinated with WH121/DMT, BCG, DMT, or PBS, respectively. The subsets of WH121-specific effector and central memory T cells were identified by intracellular cytokines staining (ICS) and multicolor flow cytometer. **A.** The absolute numbers of IFN-γ^+^, IL-2^+^ and IFN-γ^+^IL-2^+^ CD4^+^ or CD8^+^ T cells, or **B.** T_CM_ cells secreting IL-2 and T_EM_ cells secreting IFN-γ are expressed as the mean ±SD of six mice per group. *p < 0.05, 9 vs. 18 weeks. This experiment was repeated twice with similar results.

To elucidate immunological mechanisms relative to the protection, WH121 specific IFN-γ and IL-2 secreting T cells in the splenocytes were detected by multicolor flow cytometer in different vaccinated mice (Figure 5A). At 9 weeks after immunization, single IFN-γ^+^ CD4^+^ and CD8^+^ T cells were dominated in WH121/DMT vaccinated mice, while BCG vaccination mainly induced single IL-2^+^ CD4^+^ and CD8^+^ T cells. Moreover, many more single IFN-γ^+^ CD4^+^ and CD8^+^ T cells, and IFN-γ^+^IL-2^+^ CD4^+^ T cells were elicited in WH121/DMT vaccinated mice than those in the BCG group. Eighteen weeks later, single IFN-γ^+^ CD4^+^ and CD8^+^ T cells had decreased more rapidly in both groups, although WH121/DMT still had more single IFN-γ^+^ CD8^+^ T cells than the BCG vaccination. Interestingly, WH121/DMT elicited primarily IFN-γ^+^IL-2^+^ CD4^+^ and CD8^+^ T cells, and the level of IFN-γ^+^IL-2^+^ CD4^+^T cells was higher than that of BCG vaccinated mice. In addition, the number of single IL-2^+^ CD4^+^ or CD8^+^ T cells was more significantly increased (*p* < 0.05) in the BCG group, which was also much more than that in WH121/DMT vaccinated mice (*p* < 0.05).

Furthermore, IL-2 expressing T_CM_ (CD62L^hi^CD44^hi^) and IFN-γ positive T_EM_ (CD62L^lo^CD44^hi^) cells in the splenocytes between BCG and WH121/DMT vaccinated mice were also determined (Figure 5B). Many more WH121 specific IFN-γ^+^ CD4^+^ or CD8^+^ T_EM_ cells were conferred in WH121/DMT than BCG vaccinated mice at 9 weeks or 18 weeks (*p* < 0.05), although IFN-γ^+^ CD4^+^ or CD8^+^ T_EM_ cells were also decreased more significantly with time in both groups (*p* < 0.05). In contrast, IL-2^+^ CD4^+^ or CD8^+^ T_CM_ cells were increased gradually and dominated in both WH121/DMT and BCG vaccinated mice at 18 weeks (*p* < 0.05). BCG vaccine stimulated many more IL-2^+^CD8^+^ T_CM_ cells than WH121/DMT at 18 weeks (*p* < 0.05).

### Significant protection against primary infection was conferred by WH121/DMT

**Figure 6:**
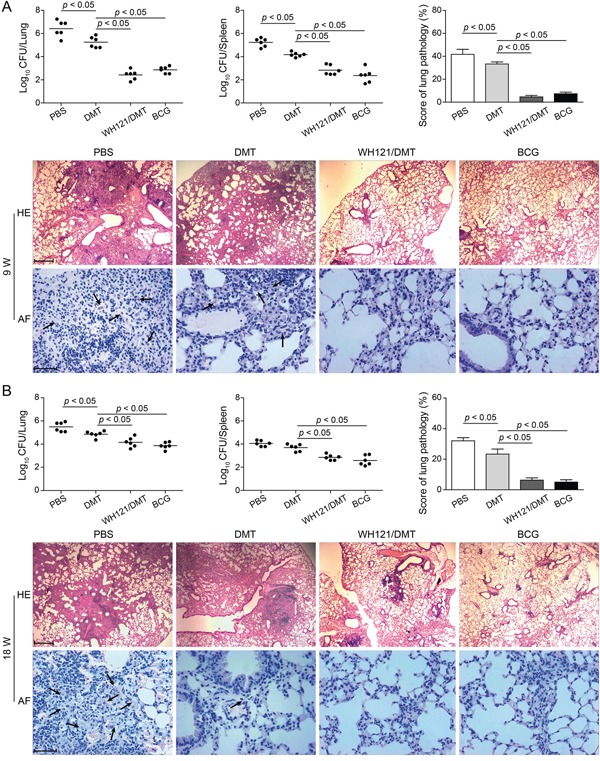
Protective efficacy of WH121/DMT vaccinated mice against primary infection C57BL/6 mice were vaccinated with WH121/DMT, BCG, DMT, or PBS. 9 **A.** or 18 **B.** weeks later, vaccinated C57BL/6 mice were challenged i.n. with 80 CFU of the *M. tuberculosis* H37Rv. Four weeks post-challenge, their lungs and spleens were harvested, and the CFU numbers per organ were counted. The results are shown as the mean of log_10_ CFU ± SEM per organ for each group (n=6) and the lung pathological scores of different vaccinated mice. The left lung lobes from different vaccinated mice were fixed and embedded for HE staining (scar bar, 400 μm) and acid-fast (AF) staining (scar bar, 50 μm). Arrows indicate AF-positive bacteria.

To evaluate the short-term and long-term protection against primary infection, vaccinated mice were challenged i.n. with approximately 80 CFU of *M. tuberculosis* at 9 (Figure 6A) and 18 (Figure 6B) weeks after immunization. Four weeks post-infection, the bacterial load in the lung and spleen and the lung pathology were compared between different groups. Of all the groups, the PBS control mice had the highest bacterial load of *M. tuberculosis* in both lung and spleen tissues during the entire experimental period (*p* < 0.05, Figure 6). Mice treated solely with DMT also experienced stronger protection than PBS controls. In particular, vaccination with BCG or WH121/DMT strongly inhibited the growth of the *M. tuberculosis* bacterial load in both organs of infected C57BL/6 mice than PBS and DMT controls at both time-points (*p* < 0.05). Most importantly, there was no statistically significant difference in lung bacterial load over time between the mice vaccinated with WH121/DMT and BCG vaccine.

The HE- and acid-fast (AF) stained lung sections showed clear differences and pathological scores between the groups (Figure 6). Acid-fast bacilli (AFB) were present throughout the all lung sections of PBS control mice, which exhibited the most serious lung pathology with extensive fibrosis, perivasculitis, and alveolitis. In contrast, lungs from mice that were vaccinated with WH121/DMT or BCG featured interstitial pneumonia that was accompanied by less pronounced lung inflammation, and far fewer AFB were observed in the tissue (Figure 6). Consistent with the results of bacterial loads observed in the lungs, scores of the lung pathology also demonstrated that WH121/DMT could provide persistent and comparable protection in the lung against primary infection, as BCG vaccine did.

### WH121/DTM enhanced the protection of BCG against the post-exposure infection

**Figure 7:**
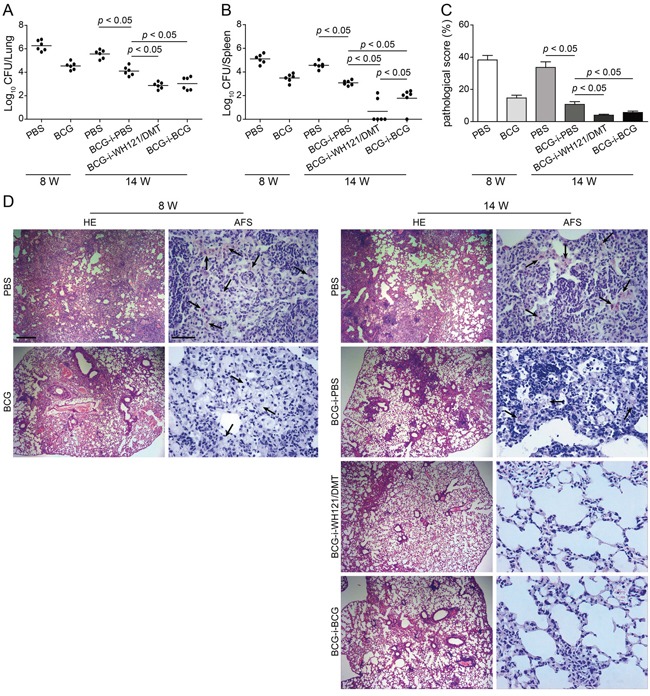
Protective efficacy of WH121 vaccinated mice against post-exposure infection Mice were first immunized s.c. with BCG or PBS, and then challenged i.n. with about 80 CFU of *M. tuberculosis* at 4 weeks after immunization. Eight weeks later, the bacterial load of both lung and spleen, and lung pathology in PBS controls and BCG vaccinated mice were compared to confirm the establishment of infection. The infected mice were further immunized with WH121/DMT, BCG or PBS. Fourteen weeks later, lungs and spleens of mice were harvested, and the CFU numbers per organ were counted. The results are shown as the mean of log_10_ CFU ± SEM per lung **A.** and spleen **B.** for each group (n=6). **C.** The lung pathological scores of different vaccinated mice. **D.** The representative lung pathology of different vaccinated C57BL/6 mice after infection. The left lung lobes from different vaccinated mice were fixed and embedded for HE staining (scar bar, 400 μm) and acid-fast (AF) staining (scar bar, 50 μm). Arrows indicate AF-positive bacteria.

To mimic the clinical progress of the post-exposure infection, mice were first immunized with BCG and then challenged i.n. with approximately 80 CFU of *M. tuberculosis* four weeks after immunization. Eight weeks later, the bacterial load of both the lung (Figure 7A) and spleen (Figure 7B), and lung pathology (Figure 7C&7D) in PBS control and BCG vaccinated mice were compared to confirm the establishment of infection. All mice after BCG vaccinated were successfully infected with *M. tuberculosis*, and at least 10^4^ CFU of bacteria were colonized in the lung, which was much lower than that in PBS controls. Then, infected mice were immunized with WH121/DMT, BCG or PBS. Fourteenth weeks later, PBS control mice still had the highest bacterial load in the lung and spleen of all groups (*p* < 0.05). Interestingly, less than 1000 CFU of *M. tuberculosis* were persistently colonized in the lung of both WH121/DMT and BCG repeat vaccinated mice, which was much lower than in only BCG primed controls (*p* < 0.05, Figure 7A). Moreover, a lower number of *M. tuberculosis* in the spleen of WH121/DMT vaccinated mice was obtained than from BCG vaccination again (*p* < 0.05). Most importantly, 4/6 of WH121/DMT group and 1/6 of BCG repeat vaccinated mice did not have detectable *M. tuberculosis* in their spleens (Figure 7B).

Consistent with the results of bacterial enumeration, PBS control mice had the most serious lung pathology, and a few AFB were found in the section of lung during the entire experimental period. In contrast, the lung pathological changes in both WH121/DMT and BCG boost groups were close to normal and few AFB was observed in the lung section of both groups.

## DISCUSSION

In this study, mice vaccinated with WH121/DMT, which consists of five antigens significantly expressed during four stages, could provide both short-term and long-term protection against *M. tuberculosis* infection, as BCG vaccinated mice did. In particular, WH121/DMT could boost the BCG primed mice against post-exposure infection and confer more significant protection than BCG alone or repeat vaccination. Therefore, our constructed WH121/DMT subunit vaccine might be an alternative to BCG vaccination in HIV infected persons for TB prevention [13].

*M. tuberculosis* can be maintained in its latent stage over an infected individual's lifetime and has the potential for reactivation as the primary source of adult TB disease, depending on the host immunity and the expression of reactivation-related genes in *M. tuberculosis* such as the resuscitation-promoting factor (Rpf) family [14]. High responders of HCs to RpfB might be attributed to the highly homologous expression of Rpf proteins between *M. tuberculosis* complex, non-tuberculosis mycobacteria and some bacterial species of other genera that may be part of the normal flora in humans [15]. In particular, the coexisting immune responses to Ag85A and Rv2626c, related with both primary and latent infection were found in both ATBs and LTBIs, which coincides with our [10] and other previous studies [16]. In addition, PhoY2 [17] and Rv3407 control the transformation process by regulating nutrition and biochemical metabolism and benefitting the persistence of *M. tuberculosis*, respectively. The IFN-γ responses to Rv3407, PhoY2, and RpfB in both ATBs and LTBIs also demonstrated for the first time that *M. tuberculosis in vivo* may change from active replication to persistence, and vice versa. Therefore, heterogenous bacteria including active growing and dormant *M. tuberculosis* might coexist in both ATBs and LTBIs and could also transfer each other *in vivo.* It also plausibly explains why the protective properties of the combined antigen WH121 are superior to those of the individual components, as demonstrated in this study. In accordance with this, the current TB chemotherapy strategy by combining multidrug targeting to address rapidly growing, persistent or dormant *M. tuberculosis* and lengthy treatments [18] has achieved very good treatment effects.

Moreover, CD4^+^ Th1-typed response has been generally accepted as the determining role in the vaccine-induced protection against TB infection [19], by the secretion of IFN-γ [20]. IFN-γ can activate macrophages to kill the intracellular *M. tuberculosis* by promoting the fusion of phagosomes and lysosomes [21] or benefiting the formation of autophagy [22]. In this study, the protection conferred by WH121/DMT might be attributed to WH121-specific CD4^+^Th1-biased responses, which are characterized by increased antigen-specific IgG2a/IgG1 ratio and high levels of IFN-γ secreted by the splenocytes of vaccinated mice. Currently, more specific biomarkers associated with the protection remain unclear. T_CM_ cells can express IL-2, multiply quickly and turn into effector T cells when they encounter the antigen again, whereas T_EM_ cells are located in infection sites and can express IFN-γ very rapidly [23]. Our study demonstrates that a significant number of IFN-γ^+^ CD4^+^ and CD8^+^ T cells, and IFN-γ^+^ T_EM_ cells are involved in vaccine-induced early protection, while the long-term protection mainly depends on the increasing IL-2^+^IFN-γ^+^CD4^+^ and CD8^+^ T cells, especially IL-2^+^ T_CM_ cells. Although BCG is an attenuated live vaccine that could express and secrete thousands of proteins, it could not confer a significant immune response to latent antigens [16]. Therefore, WH121/DMT also elicits a stronger WH121 specific Th1-typed T cell immunity than BCG vaccinated mice. In a recent clinical trial, MVA85A expressing Ag85A, the single-stage antigen of active replication phase, as a booster in BCG-primed infants show no improved protection by BCG against TB [24]. In contrast, the cocktail rBCG strains [25], the single BCG strain [12] expressing two-stage antigens, or the two stage subunit vaccine candidates [6, 8, 26] could enhance the protection of BCG in mice.

In conclusion, multistage antigens are the promising target for the development of the next generation TB vaccine. Screening much more protective antigens expressed from different stages of *M. tuberculosis* is a very important task that might be beneficial for improving the protection of vaccine candidates. The strategy by combining many more stage-specific antigens of *M. tuberculosis* expressed during primary infection, latency and reactivation, to induce the preexisting immunity targeting coexisting status of *M. tuberculosis* after infection *in vivo*, might provide significant protection against pre- and post-exposure infection, and WH121/DMT for further preclinical evaluation is warranted.

## MATERIALS AND METHODS

### Ethics statement

The study protocol involving human subjects was approved by the Ethics Committee of Tongji Medical College (Wuhan, China), and written informed consent was obtained from all recruited subjects at the beginning of the experiments. Human experiments were performed in accordance with approved guidelines of Tongji Medical College. Mouse experiments were performed in accordance with the guidelines of the Chinese Council on Animal Care and approved by both Tongji School Committee on Biosafety and the Ethics Committee of Animal Experimentation of Tongji Medical College.

### Prokaryotic expression and purification of WH121

The DNA sequence of the fusion protein WH121 was designed by linking five genes of *M. tuberculosis* H37Rv; namely, Rv3407, PhoY2, Rv2626c, Ag85A, and RpfB and cloned into the commercially synthesized plasmid pDC316-WH121 (Life Invitrogen, Shanghai, China). The coding sequence of WH121 and genes encoding the multistage antigens Rv3407, PhoY2, Rv2626c, and RpfB were amplified by PCR with their respective primers and conditions (Supplementary Table 1). All the PCR products were cloned into the *Nde* I and *Xho* I sites of the pET-30b prokaryotic expression vector. The recombinant plasmids were transformed into *E. coli* BL21(DE3) strains and were induced by incubating them with isopropylthio-β-D-galactoside (IPTG) at a final concentration of 1 mM for 4 h. Purification was performed by using NTA-metal ion affinity chromatography (GE Healthcare, Somerset, NJ, USA). Recombinant proteins were dialyzed against sterilized PBS with a urea concentration gradient (from 8 M to 0 M) for 48 h at 6 h intervals. The proteins were lyophilized, diluted in phosphate-buffered saline (PBS) by using pyrogen-free reagents, aliquoted, and stored at -20°C. The residual endotoxin contamination was verified to be below 0.1 EU/mL of protein. The protein concentration was determined using a BCA Protein Assay Kit (Beyotime, Shanghai, China). The purified recombinant proteins were monitored by 15% SDS-PAGE.

### Immunogenicity of WH121 and multistage antigens in human TB infections

ATBs and LTBIs were enrolled from the Xinxiang Institute of TB Prevention and Treatment (Xinxiang, Henan Province, China), and non-infected HCs were recruited from Tongji Medical College. The enrollment form including purpose and content of the experiment was first distributed to each participant, and then the demographic information of consenting participants was collected through face-to-face questionnaire investigations. All participants were further screened using the diagnostic standards (Supplementary Table 2), and all subjects were seronegative for HIV infection. A recombinant CFP21-MPT64-whole blood IFN-γ assay (rCM-WBIA) [27] was further used to confirm the rCM-WBIA-positive ATBs and LTBIs, and the rCM-WBIA negative HCs, to avoid any effects from the BCG vaccination on the diagnostic results. Before the treatment, 5 mL of freshly heparinized whole blood was collected from each donor, and an antigen-specific whole blood IFN-γ assay was performed and analyzed as previously described [10].

### Immunization of mice

The adjuvant DMT was prepared as previously described [11]. Each dose of the mixture contained 20 μg/100 μL of each protein emulsified in 100 μL of DMT adjuvant. For the immunization, specific-pathogen-free female C57BL/6 (H-2^b^) mice at 6 weeks of age were purchased from the Center for Animal Experiment of Wuhan University (Wuhan, China) and maintained in a biosafety laboratory on standard laboratory chow. To compare the protection between different antigens, the mice were immunized subcutaneously (s.c.) once with 0.2 mL of each antigen/DMT mixture. To confirm the immunogenicity and protection against primary and post-exposure infection, the mice were immunized subcutaneously (s.c.) twice with 0.2 mL of WH121/DMT at 3-week intervals. The BCG China was used as a positive control and vaccinated s.c. at the proximal end of the tail with approximately 1×10^6^ CFU in a final volume of 200 μL of PBS. Control mice were treated with 200 μL of PBS. The detail regimens are shown as Supplementary Figure 1.

### WH121-specific antibody titers

9 and 18 weeks after immunization, the WH121-specific antibodies IgG, IgG1, and IgG2a (which was replaced by IgG2c) in the serum of each mouse from different vaccinated groups were assessed by ELISA as previously described [10], and the results are expressed as the mean (±SEM) log_10_ endpoint titers per group (n=6).

### WH121-specific IFN-γ secreted by splenocytes

At 9 and 18 weeks after immunization, the spleen from each mouse in different groups was aseptically removed. The spleen cells were prepared, counted, and seeded in triplicates at 2.5 × 10^6^ cells/well in a 24-well plate and incubated with WH121 (10 μg/mL) at 37°C under 5% CO_2_. Seventy-two hours later, the culture supernatants were harvested for IFN-γ assay by using double-sandwich ELISA kits (Multi Sciences LTD., Hangzhou, China) as previously described [10]. The levels of antigen-specific cytokines are expressed as the mean ± SD (pg/mL) for each group (n=6).

### WH121-specific memory T cells analysis

Spleen cells were further seeded in triplicates at 5 × 10^6^ cells/well in a 24-well plate and stimulated with WH121 (10 μg/mL) in the presence of 1 μg/mL anti-CD28/CD49d (eBioscience CA, USA) for 20 h at 37°C under 5% CO_2_. For the last 4 h of the stimulation, 3 μg/mL brefeldin A and 2 μM monensin solution (eBioscience CA, USA) were added. The cells were washed in FACS buffer (1% FCS-PBS) and stained for 30 min at RT with anti-CD4-PE-Cy7 (clone GK1.5, eBioscience), anti-CD8α-PE (clone53-6.7, eBioscience), anti-CD62L-FITC (clone MEL-14, BD Pharmingen), and anti-CD44-APC-Cy7 (clone IM7, BD Pharmingen) mAbs. After the cells had been washed and permeabilized with the Cytofix/Cytoperm kit (BD Pharmingen CA, USA), the intracellular cytokines were stained with anti-IFN-PerCP-Cy5.5 (clone XMG1.2; eBioscience CA, USA) and anti-IL-2-allophycocyanin (clone JES6-5H4; eBioscience CA, USA) mAbs for 30 min at RT. The cells were washed twice, resuspended in FACS buffer and analyzed using an LSRII multicolor flow cytometer (BD Biosciences CA, USA). The absolute number of IFN-γand/or IL-2 positive CD4^+^ and CD8^+^ T cells, T_CM_ (central memory T cells, CD62L^hi^CD44^hi^) and T_EM_ (effector memory T cells, CD62L^lo^CD44^hi^) cells were analyzed with FlowJo software (Tree Star Inc., OH, USA) as previously described [25], respectively. The data are represented as the mean ± SD per group (n = 6).

### Protection evaluation against primary infection

Six weeks after immunization, DMT adjuvanted multistage antigens or WH121 vaccinated mice were challenged i.n. with the virulent *M. tuberculosis* H37Rv strain to compare the protection between different antigens. Both PBS and DMT were used as negative controls. On the next day, three mice in the PBS control group were killed and the entire lung was removed aseptically for the enumeration of *M. tuberculosis* to determine the actual infection dose. Four weeks post-challenge, the lung from each mouse was removed to assess the efficacy of the immunization, which was determined by comparing the bacterial load in lung (n=6) [12].

For the protection evaluation of WH121/DMT, mice in each group were separately challenged i.n. with *M. tuberculosis* 9 and 18 weeks after immunization. Four weeks post-challenge, mice were euthanized to assess the immunization's protective efficacy, which was determined by comparing the bacterial load in the spleen and lung (n=6), and by observing the lung histopathological changes as previously described (n=3) [12].

### Protection evaluation against post-exposure infection

The *M. tuberculosis* post-exposure infection model was established as previously described procedures with a few modifications [28]. Briefly, mice were first vaccinated s.c. with BCG once while control mice were treated with PBS. Four weeks later, all mice were challenged i.n. with virulent *M. tuberculosis* H37Rv strain. Eight weeks after initial vaccination, BCG vaccinated mice were randomly divided into three groups (six mice in each group) and then vaccinated with WH121/DMT, BCG and PBS. The dose and protocol of each immunization was performed as previously described. Fourteen weeks later, lungs and spleens of mice were harvested and compared efficacy between groups.

### Statistical analysis

The antigen-specific IFN-γ levels in the different groups of human subjects were analyzed with a nonparametric Mann-Whitney *U*-test. Student *t* test was used to compare the other biomarkers among different experiment groups of mice. A statistical analysis was performed with SPSS 17.0 software, and the difference was considered significant when *p* < 0.05.

## SUPPLEMENTARY FIGURE AND TABLES






