# Supplementary material for: Mycobacterium tuberculosis multistage antigens confer comprehensive protection against pre- and post-exposure infections by driving Th1-type T cell immunity
Source: Oncotarget. 2016 Aug 23;7(39):63804–15. doi: 10.18632/oncotarget.11542 (PMC5325405; doi:10.18632/oncotarget.11542)
Supplement: Supplementary file 1 [file oncotarget-07-63804-s001.pdf]

Mycobacterium tuberculosis multistage antigens confer comprehensive protection against pre- and post-exposure infections by driving Th1-type T cell immunity

SUPPLEMENTARY FIGURE AND TABLES

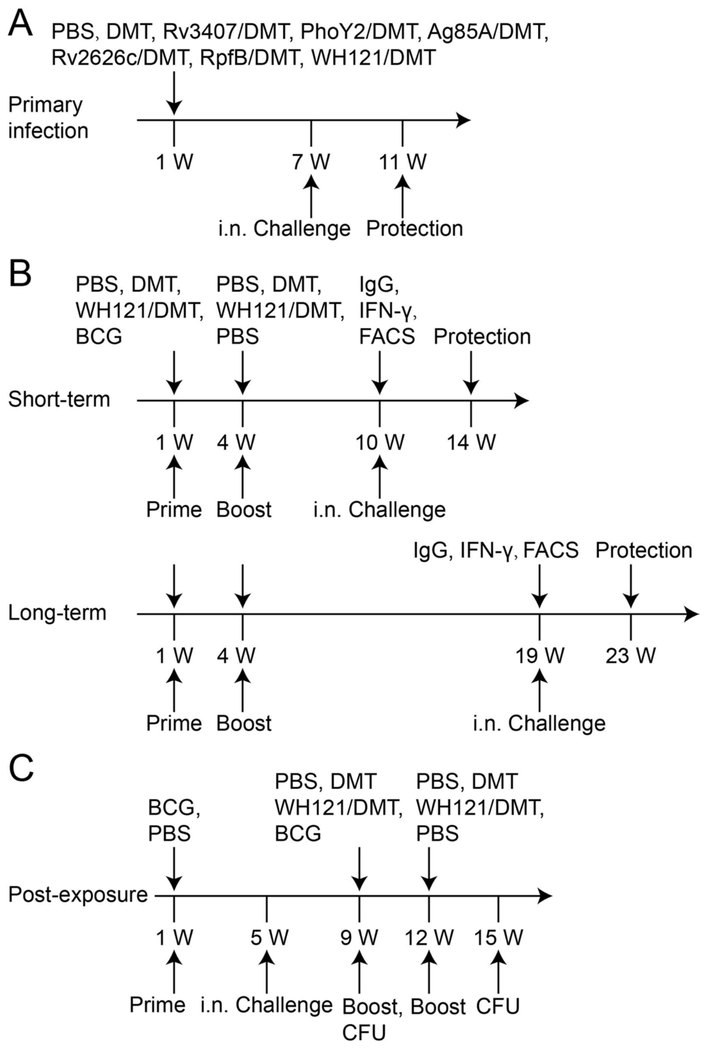

Supplementary Figure S1: Immunization scheme for 3 regimens. A. WH121 and multistage antigen protection in a primary infection regimen. **B.** Short-term and long-term protective efficacy of WH121/DMT against primary infection regimen. **C.** Protective efficacy of WH121 against post-exposure infection regimen.

Supplementary Table S1: Primers and thermal cycle parameters for the cloning of *M. tuberculosis* antigens

See Supplementary File S1.

Supplementary Table S2: Characteristics of human subjects

See Supplementary File S2.
